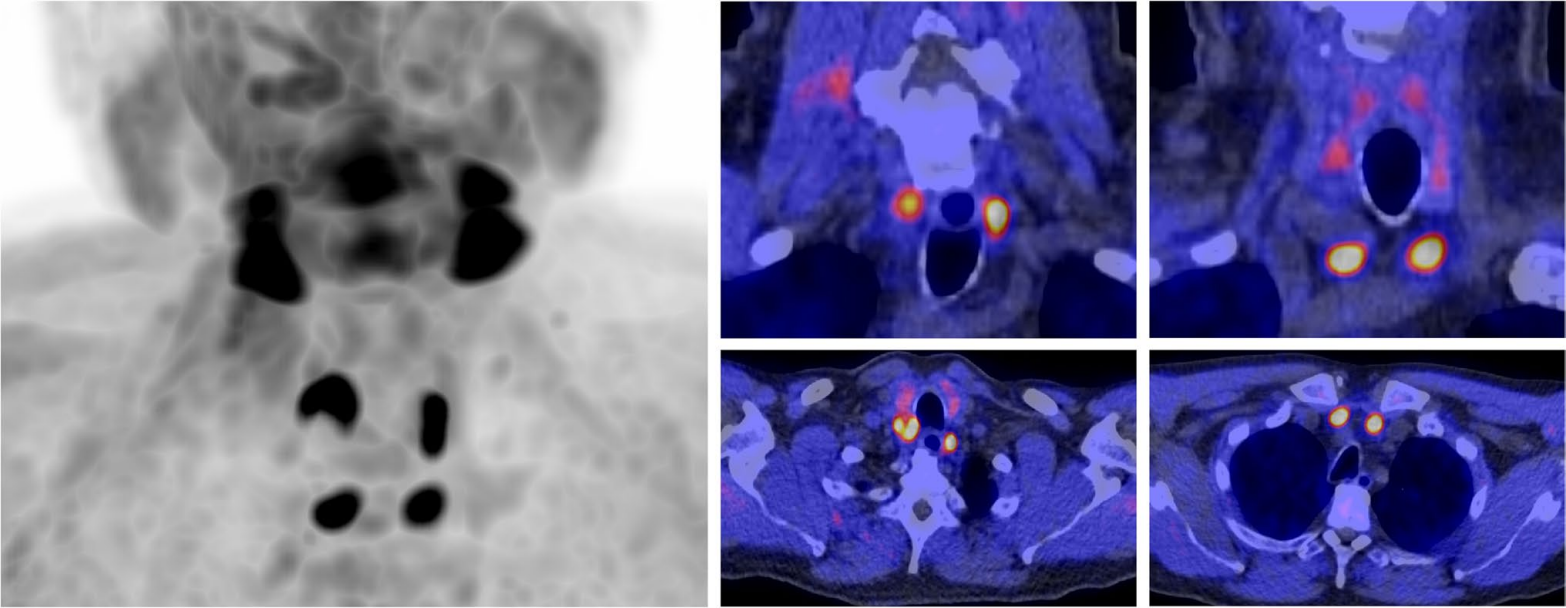# Use of [^11^C]choline PET/CT for visualization of four hyperactive parathyroid glands in a patient with renal hyperparathyroidism

**DOI:** 10.1007/s00259-022-05885-8

**Published:** 2022-06-28

**Authors:** Matti Raitza, Aziz A. N. Alshalali, Andor W. J. M. Glaudemans, Rijk O. B. Gans, Riemer H. J. A. Slart

**Affiliations:** 1 Rostock University Medical Center, University of Rostock, Rostock, Germany; 2 Medical Imaging Center, Department of Nuclear Medicine and Molecular Imaging, University of Groningen, University Medical Center Groningen, Hanzeplein 1, 9700 RB Groningen, The Netherlands; 3 Department of Internal Medicine, University Medical Center Groningen, University of Groningen, Groningen, The Netherlands; 4 Faculty of Science and Technology, Department of Biomedical Photonic Imaging, University of Twente, Enschede, The Netherlands

A 62-year-old male patient with a history of renal phosphaturia and slowly progressive chronic kidney disease (CKD) G4A2H presented with fatigue and hypercalcemia (2.7 mmol/L corrected for albumin) with an elevated parathyroid hormone (507 pg/mL).

He underwent a [^11^C]choline PET/low dose CT (20 min post-injection 440 MBq) to assess and differentiate more precisely the anatomical and functional presence of parathyroid hyperfunction.

The scan shows an intense focal [^11^C]choline uptake in the area of each of the four parathyroid glands (left MIP, see figure), with moderate higher levels in the upper two parathyroid glands (SUVmax 10.4) (middle column figure, and visible as well on the maximum intensity projection (MIP) image on the left side) in comparison to the lower two (SUVmax 8.7) (right-sided in figure). Based on this distribution pattern and the context of CKD, a diagnosis of secondary hyperparathyroidism with parathyroid hyperplasia was made, as well as tertiary (or primary, but less likely) hyperparathyroidism considering the high uptake in all parathyroid glands and the elevated serum calcium levels which is an unlikely finding in CKD. He was successfully treated with cinacalcet 60 mg bid.

Recently, radiolabeled choline, both in the form of [^11^C]choline and [^18^F]fluorocholine, has emerged as a highly specific and sensitive PET tracer for the identification of parathyroid adenomas [1–3]. [^18^F]fluorocholine has been studied much more extensively, because its longer half-life facilitates transport when no cyclotron is available on site. PET imaging is predominantly used to detect active parathyroid adenoma for the diagnosis of primary hyperparathyroidism, but the value of choline PET imaging for diagnosing secondary and tertiary hyperparathyroidism is largely unknown. Studies have shown that preoperative planning with nuclear imaging reduces the extent of surgical resection as well as the rate of surgical failure due to missing ectopic parathyroid glands [4, 5]. If in our case poor medical drug response should be noted, the three most hyperactive parathyroid glands would be selectively removed by the surgeon based on the choline PET imaging findings [6, 7]. Imaging directed based surgery reduces redo-surgeries and the perioperative complication rate will be very low [6]. Successful preoperative localization of enlarged and hyperfunctioning parathyroids increases cure rates [6].

[^11^C]choline or potential [^18^F]choline PET therefore appears to be a promising tracer for the differentiation between the primary, secondary, and tertiary hyperparathyroidism and may have consequences for treatment planning.